# Depressive symptoms and metabolic syndrome components among older Chinese adults

**DOI:** 10.1186/s13098-020-00526-2

**Published:** 2020-02-18

**Authors:** Jing-Hong Liu, Yu-Xi Qian, Qing-Hua Ma, Hong-Peng Sun, Yong Xu, Chen-Wei Pan

**Affiliations:** 1 School of Public Health, Medical College of Soochow University, 199 Ren Ai Road, Suzhou, 215123 China; 2 The 3rd People’s Hospital of Xiangcheng District, Suzhou, China

**Keywords:** Depressive symptoms, Metabolic syndrome components, Public health, Epidemiology

## Abstract

**Background:**

Few studies examined associations between depressive symptoms and metabolic syndrome (MetS) among older Chinese adults. Considering that the prevalence of depressive symptoms is high in older Chinese adults, we aimed to examine associations of depressive symptoms with MetS and its components in older Chinese adults.

**Methods:**

Data from a community-based cross-sectional study of 4579 Chinese adults aged 60 years or older were analyzed. Depressive symptoms were assessed using the nine-item Patient Health Questionnaire. The presence of MetS was defined based on the Adult Treatment Panel III criteria, which include obesity, reduced blood high-density lipoprotein, high blood pressure (BP), elevated fasting plasma glucose and hypertriglyceridemia. A participant was considered as having MetS if he or she met at least three of the above-mentioned criteria.

**Results:**

In all participants, depressive symptoms were related to elevated fasting plasma glucose (≥ 7.0 mmol/L) (adjusted odds ratio [OR] = 1.50, 95% confidence interval [CI] [1.00–2.20]) and diabetes (adjusted OR = 1.50, 95% CI [1.01–2.20]). The associations of depressive symptoms with MetS and its components were not significant among women. However, there was a negative association between depressive symptoms and elevated systolic BP (≥ 130 mm Hg) (OR = 0.59, 95% CI [0.4–0.9]), and similar findings were observed after adjusting for lifestyle-related variables in men.

**Conclusions:**

In older Chinese adults, depressive symptoms were negatively associated with elevated systolic BP in men while these findings were not found in women.

## Background

The World Health Organization has estimated that more than 300 million people worldwide suffered from depression in the year 2015 [1]. Depression is a major mental health problem in old people. Population-based prevalence of depression in seniors was about 4.2–25.1% as reported in different studies [2–5]. Individuals with depressive symptoms may be also susceptible to metabolic syndrome (MetS) which is a common health issue in older adults. The potential mechanism might be related to its co-occurrence with obesity [6], as obesity is an important component of MetS and visceral adipose tissue secretes inflammatory cytokines that could cause chronic inflammation, and trigger MetS [7–9]. In addition, gender may be an effect modifier in the relationship between depression or depressive symptoms and MetS, even though studies reported inconsistent findings regarding the moderation effect of gender on the association between depression and MetS [10–12]. For example, a longitudinal study in Europe indicated that women but not men with depressive symptoms are vulnerable to MetS [10, 11]. On the contrary, a study from Japan found that the association only presented in men [12]. But a study conducted in Norway found none of the interaction effects related to gender [13].

Depression is a mental health problem affected by various psychosocial factors, findings from a single study conducted in a specific area can not be extrapolated to other populations with different age, culture and ethnicity. The prevalence of depressive symptoms is high in older Chinese adults [14]. But few studies assessed its association with MetS among this population. Thus, this study aimed to assess the association of depressive symptoms with MetS in Chinese adults aged 60 years or older in a community-based study.

## Methods

### Study design and procedure

**Fig. 1:**
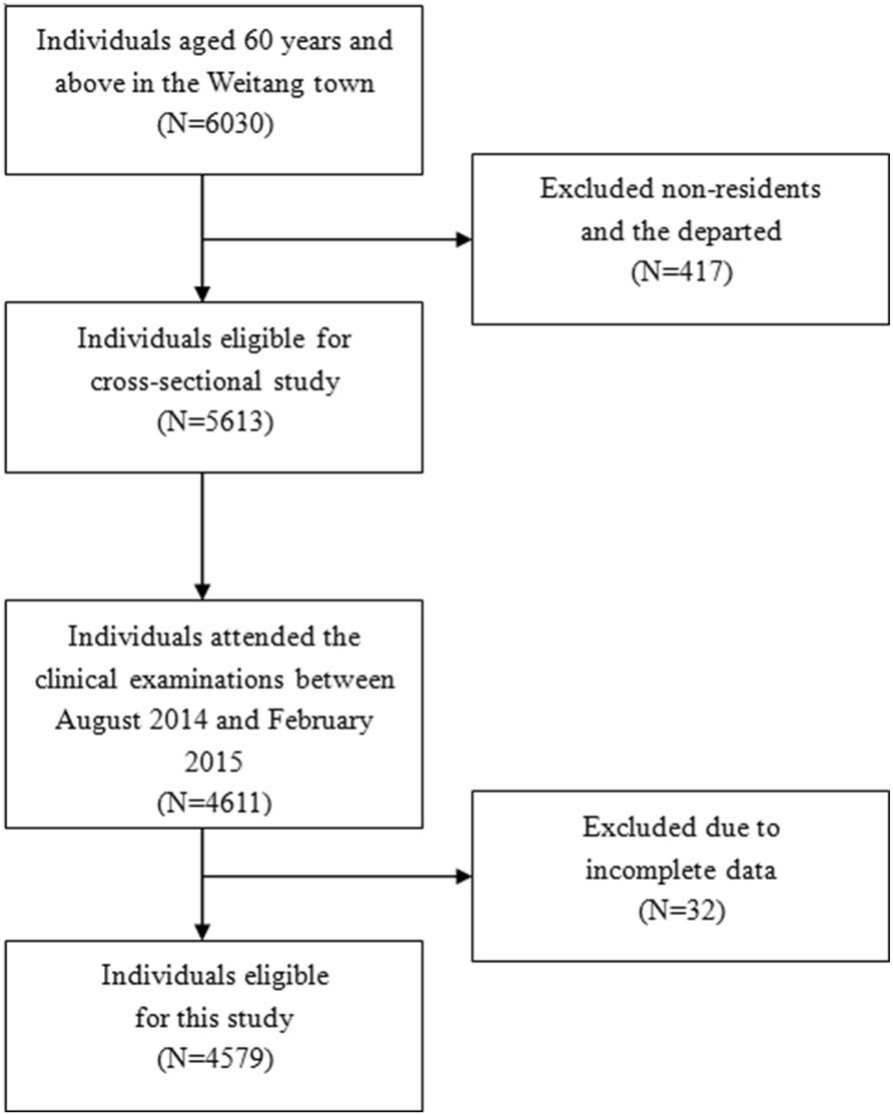
Flow-chart for sample construction of the cross-sectional study

Weitang Geriatric Diseases study is a community-based study conducted in the Weitang town among adults aged 60 years or older in Suzhou located in the east part of China. Detailed study protocol has been described elsewhere [15, 16]. Briefly, participants were screened based on local official records and those (n = 6030) aged 60 years or older were invited via sent-home letters. Exclusion criteria applied to those whom had migrated from the residing address, had been living there shorter than 6 months, or deceased. From August 2014 to February 2015, a total of 5613 adults enrolled and 4611 attended the clinical examinations. (Fig. 1) The final sample consisted of 4579 participants with completed data from anthropometric examinations and blood sample analyses.

The study was conducted in accordance with the tenets of the Helsinki Declaration and was approved by the Institutional Review Board of Soochow University.

### Assessment of depressive symptoms

Depressive symptoms were measured using the nine-item Patient Health Questionnaire (PHQ-9), which is a validated instrument with high sensitivity and specificity [17, 18]. The PHQ-9 consists of nine items and each item corresponds to one criterion of the fourth edition of Diagnostic and Statistical Manual of Mental Disorders diagnosis for symptoms of major depression [18]. Eight items of the PHQ-9 are about the frequency of depressive symptoms, and the last item is about the frequency of thinking about hurting themselves during the past two weeks. Item responses were based on a 4-point Likert scale of “not at all”, “several days”, “more than half the days”, and “nearly every day” with the scores of 0, 1, 2, and 3, respectively. Participants with total scores ranging from 5 to 27 were regarded as having depressive symptoms [18, 19].

### Assessment of MetS and its components

Body weight was measured in kilograms with light clothing using a digital scale. Height was measured in centimetres without shoes using a wall-mounted measurement tape. Body mass index (BMI) was calculated as the weight in kilograms divided by the square of the height in meters. Blood samples were collected and analyzed with the standard laboratory assays to obtain high-density lipoprotein (HDL) cholesterol, fasting plasma glucose (FPG) and blood triglycerides. Blood pressure (BP) was measured at least 3 times after 5 min or longer intervals of rest with an automatic blood pressure monitor (Dinamap model Pro Series DP110X-RW, 100V2; GE Medical Systems Information Technologies, Inc., Milwaukee, Wisconsin, United States). The value of BP was calculated from the average of the last two readings. The definition of MetS was based on the National Cholesterol Education Program-Adult Treatment Panel III [20]. A participant was considered as having MetS if he or she met at least three of the following criteria: (a) abdominal obesity defines as BMI of 25 kg/m^2^ or higher [21], (b) HDL cholesterol lower than 40 mg/dL for men when 50 mg/dL for women, (c) BP of 130/85 mmHg or above or use of antihypertensive medications, (d) FPG of 7.0 mmol/L or greater or with physician-diagnosed diabetes mellitus, (e) blood triglycerides defines as 150 mg/dL (1.7 mmol/L) or above.

### Assessment of covariates

The data of social-demographic characteristics (i.e. age, gender, education level, marital status) was collected by trained interviewers using a pre-designed questionnaire. Marital status was divided into living “with” and “without” spouse, and education level was classified into “primary education or below” and “secondary education or above”. Lifestyle-related variables including smoking (current/former/never), alcohol drinking (drinkers/non-drinkers), tea consumption (drinkers/non-drinkers), dietary pattern (normal/vegetarian) and sleep quality (good/intermediate/poor) were self-reported. Information regarding medication intake were collected and no participants had used antidepressant medications before.

### Statistical analysis

To compare the characteristics of participants with and without depressive symptoms, Chi square test and student’s t-test were used for categorical and continuous variables, respectively. Associations of depressive symptoms with MetS and its components were assessed using logistic regression models. All models were established separately for MetS and its individual components (i.e. BMI ≥ 25 kg/m^2^, systolic BP (SBP) ≥ 130 mm Hg, diastolic BP (DBP) ≥ 85 mm Hg, blood triglycerides ≥ 1.7 mmol/L, HDL cholesterol < 1.0 in men or < 1.3 mmol/L in women, FPG ≥ 7.0 mmol/L, diabetes). Model 1 did not adjust for any covariates. Model 2 adjusted for social-demographic characteristics (age, gender, education level, and marital status). Model 3 additionally adjusted for lifestyle habits (smoking, alcohol consumption, tea consumption, dietary pattern, and sleep quality). In addition, gender-stratified analyses were performed to examine the interaction effects of gender.

All statistical analyses were carried out using the SPSS version 21.0 (SPSS Inc., Chicago, IL, USA), and a P value of less than 0.05 was considered statistically significant.

## Results

The mean age of the participants included in this analysis was 67.4 ± 6.3 years old. Individuals with depressive symptoms accounted for 8% of the whole population and women were more likely to have depressive symptoms (P < 0.001). The characteristics of participants with and without depressive symptoms have been reported in a previous publication [22]. Table 1 shows comparisons of participants’ statuses of MetS and its components by the presence of depressive symptoms in the overall sample and in males and females, separately. Overall, 20.0% of the participants without depressive symptoms and 20.8% with depressive symptoms were affected by MetS and the difference was not statistically significant (P = 0.70). With regards to the specific components of MetS, we found that participants with depressive symptoms tended to have lower BMI (P<0.001) and lower DBP (P = 0.04). The distributions of other MetS components between participants with and without depressive symptoms were not significantly different. Gender-stratified analyses indicated that depressive women were more likely to have lower BMI (P = 0.001), and depressive men tended to have lower DBP (P = 0.02) and lower triglycerides (P = 0.012) compared with their non-depressive counterparts.

**Table 1:** Metabolic syndrome characteristics of study participants by gender and depressive symptoms

	Total			Women			Men		
	No depressive symptoms (n = 4214)	Depressive symptoms (n = 365)	P	No depressive Symptoms (n = 2124)	Depressive symptoms (n = 255)	P	No depressive symptoms (n = 2090)	Depressive symptoms (n = 110)	P
Metabolic syndrome, n (%)	843 (19.99)	76 (20.82)	0.70	548 (25.77)	60 (23.53)	0.44	295 (14.12)	16 (14.55)	0.90
High BMI^a^, n (%)	825 (19.59)	59 (15.89)	0.09	382 (17.99)	37 (14.51)	0.17	444 (21.20)	21 (19.09)	0.59
High BP^b^, n (%)	3604 (85.54)	326 (89.32)	0.047	1863 (87.71)	234 (91.76)	0.058	1741 (83.34)	92 (83.63)	0.94
High triglycerides^c^, n (%)	931 (22.08)	80 (21.92)	0.94	561 (26.38)	63 (24.71)	0.57	370 (17.71)	17 (15.45)	0.55
Low HDL^d^, n (%)	965 (22.89)	87 (23.84)	0.68	720 (33.88)	74 (29.02)	0.12	245 (11.73)	13 (11.82)	0.98
Glucose component^e^, n (%)	462 (10.97)	50 (13.70)	0.11	237 (11.16)	33 (12.94)	0.40	225 (10.77)	17 (15.45)	0.13
BMI, mean (SD), kg/m^2^	23.32 (2.61)	22.81 (2.82)	< 0.001	23.26 (2.61)	22.66 (2.89)	0.001	23.39 (2.62)	23.14 (2.65)	0.33
Fasting glucose, mean (SD), mmol/L	5.58 (1.10)	5.68 (1.57)	0.25	5.64 (1.11)	5.65 (1.35)	0.86	5.53 (1.08)	5.75 (1.99)	0.26
Systolic BP, mean ± SD, mm Hg	144.24 (19.75)	145.14 (18.68)	0.41	147.55 (19.75)	148.02 (18.19)	0.71	140.89 (19.20)	138.45 (18.14)	0.19
Diastolic BP, mean ± SD, mm Hg	85.83 (11.18)	84.57 (11.49)	0.04	85.35 (10.81)	84.97 (11.56)	0.60	86.31 (11.53)	83.64 (11.31)	0.02
Triglycerides, mean ± SD, mmol/L	1.36 (0.81)	1.33 (0.77)	0.57	1.45 (0.78)	1.42 (0.84)	0.57	1.27 (0.83)	1.13 (0.51)	0.012
HDL, mean ± SD, mmol/L	1.46 (0.39)	1.49 (0.41)	0.09	1.48 (0.37)	1.51 (0.38)	0.23	1.43 (0.40)	1.46 (0.48)	0.58
Diabetes, n (%)	329 (7.81)	38 (10.41)	0.08	176 (8.29)	26 (10.20)	0.30	153 (7.32)	12 (10.91)	0.16
Antihypertensive medication, n (%)	2174 (51.61)	199 (54.52)	0.29	1110 (52.28)	138 (54.12)	0.58	1064 (50.93)	61 (55.45)	0.36

*BMI* body mass index; *BP* blood pressure; *HDL* high-density lipoprotein; *SD* standard deviation

^a^ BMI ≥ 25 kg/m^2^

^b^Systolic BP ≥ 130 mm Hg or diastolic BP ≥ 85 mm Hg or antihypertensive medication

^c^ Blood triglycerides ≥ 1.7 mmol/L

^d^ HDL cholesterol < 1.0 mmol/L in men and  < 1.3 mmol/L in women

^e^ Fasting plasma glucose ≥ 7.0 mmol/L or with a history of diabetes

Table 2 shows the effect estimates (odds ratios, ORs) together with the 95% confidence intervals (CIs) on the associations of depressive symptoms with MetS and its individual component. We found that depressive symptoms were negatively related to elevated DBP (≥ 85 mm Hg) (crude OR = 0.77, 95% CI [0.6–1.0]) but positively related to elevated FPG (≥ 7.0 mmol/L) (crude OR = 1.47, 95% CI [1.03–2.1]). After adjusting for age, gender, education level and marital status, the associations between MetS and depressive symptoms were no longer significant except for the association with elevated FPG (OR = 1.47, 95% CI [1.01–2.1]). This association remained significant even after additionally controlling for other potential confounders (smoking, alcohol consumption, dietary pattern and sleep quality) (OR = 1.50, 95% CI [1.00–2.2]). Furthermore, diabetes was related to the presence of depressive symptoms (OR = 1.50, 95% CI [1.01–2.2]).

**Table 2:** Associations of depressive symptoms with metabolic syndrome and its individual component

	Depressive symptoms	
	OR (95% CI)	P
Metabolic syndrome		
Model 1	1.05 (0.80–1.40)	0.70
Model 2	1.03 (0.80–1.30)	0.86
Model 3	1.02 (0.80–1.30)	0.87
BMI ≥ 25 kg/m^2^		
Model 1	0.78 (0.60–1.00)	0.09
Model 2	0.95 (0.70–1.30)	0.75
Model 3	0.97 (0.70–1.30)	0.86
Systolic BP ≥ 130 mm Hg		
Model 1	1.17 (0.90–1.50)	0.25
Model 2	0.84 (0.60–1.10)	0.22
Model 3	0.87 (0.60–1.20)	0.34
Diastolic BP ≥ 85 mm Hg		
Model 1	0.77 (0.60–1.00)	0.01
Model 2	0.83 (0.70–1.00)	0.09
Model 3	0.94 (0.70–1.20)	0.61
Blood triglycerides ≥ 1.7 mmol/L		
Model 1	0.99 (0.80–1.30)	0.94
Model 2	0.99 (0.80–1.30)	0.92
Model 3	0.98 (0.70–1.30)	0.87
HDL < 1.0 (men) or < 1.3 mmol/L (women)		
Model 1	1.05 (0.80–1.40)	0.68
Model 2	0.90 (0.70–1.20)	0.42
Model 3	0.89 (0.70–1.20)	0.40
Fasting plasma glucose ≥ 7.0 mmol/L		
Model 1	1.47 (1.03–2.10)	0.04
Model 2	1.47 (1.01–2.10)	0.04
Model 3	1.50 (1.00–2.20)	0.05
Diabetes		
Model 1	1.37 (0.96–2.00)	0.08
Model 2	1.41 (0.98–2.00)	0.06
Model 3	1.50 (1.01–2.20)	0.05

*HDL* high-density lipoprotein, *BMI* body mass index, *BP* blood pressure

*OR* odds ratio, *CI* confidence interval

Model 1, crude model

Model 2 adjusted for age, gender, education level, marital status

Model 3 adjusted for model 2 plus smoking, alcohol consumption, tea consumption, dietary pattern, sleep quality

Gender might be a possible effect modifier on the relationship between depressive symptoms and DBP (P = 0.026). The gender-specific associations are shown in Table 3. Among women, none of the MetS individual component was related to depressive symptoms. Among men, depressive symptoms were negatively associated with elevated DBP in model 1 (crude OR = 0.58, 95% CI [0.4–0.85]) and model 2 (OR = 0.66, 95% CI [0.4–0.98]), but not in model 3. Additionally, depressive symptoms was associated with elevated SBP (≥ 130 mm Hg) among men in model 2 and 3 (OR = 0.59, 95% CI [0.4–0.9]) but not in model 1.

**Table 3:** Associations of depressive symptoms with metabolic syndrome and its individual component by gender

	Depressive symptoms			
	Men		Women	
	OR (95% CI)	P	OR (95% CI)	P
Metabolic syndrome				
Model 1	1.04 (0.60–1.80)	0.90	0.89 (0.70–1.20)	0.44
Model 2	1.30 (0.70–2.20)	0.35	0.95 (0.70–1.30)	0.74
Model 3	1.49 (0.80–2.70)	0.19	0.85 (0.60–1.20)	0.36
BMI ≥ 25 kg/m^2^				
Model 1	0.88 (0.50–1.40)	0.60	0.77 (0.50–1.10)	0.17
Model 2	1.06 (0.60–1.70)	0.82	0.90 (0.60–1.30)	0.59
Model 3	1.27 (0.70–2.20)	0.37	0.96 (0.60–1.40)	0.85
Systolic BP ≥ 130 mm Hg				
Model 1	0.72 (0.50–1.10)	0.11	1.34 (0.90–1.90)	0.12
Model 2	0.59 (0.40–0.90)	0.01	1.08 (0.70–1.60)	0.70
Model 3	0.59 (0.40–0.90)	0.02	1.14 (0.80–1.70)	0.54
Diastolic BP ≥ 85 mm Hg				
Model 1	0.58 (0.40–0.85)	<0.01	0.89 (0.70–1.20)	0.40
Model 2	0.66 (0.40–0.98)	0.04	0.92 (0.70–1.20)	0.52
Model 3	0.77 (0.50–1.16)	0.22	1.03 (0.80–1.40)	0.83
Blood triglycerides ≥ 1.7 mmol/L				
Model 1	0.85 (0.50–1.40)	0.55	0.99 (0.80–1.30)	0.94
Model 2	1.05 (0.60–1.80)	0.85	0.96 (0.70–1.30)	0.79
Model 3	1.04 (0.60–1.90)	0.88	0.92 (0.70–1.20)	0.57
HDL < 1.0 (men) or < 1.3 mmol/L (women)				
Model 1	1.01 (0.60–1.80)	0.98	0.80 (0.60–1.10)	0.12
Model 2	1.24 (0.70–2.20)	0.49	0.84 (0.60–1.10)	0.23
Model 3	1.21 (0.60–2.30)	0.56	0.83 (0.60–1.10)	0.23
Fasting plasma glucose ≥ 7.0 mmol/L				
Model 1	1.67 (0.90–3.10)	0.09	1.42 (0.90–2.20)	0.13
Model 2	1.63 (0.90–3.00)	0.12	1.39 (0.90–2.20)	0.17
Model 3	1.48 (0.80–2.90)	0.26	1.56 (0.90–2.60)	0.09
Diabetes				
Model 1	1.55 (0.80–2.90)	0.17	1.26 (0.80–1.90)	0.30
Model 2	1.60 (0.80–3.00)	0.15	1.33 (0.90–2.10)	0.21
Model 3	1.56 (0.80–3.10)	0.21	1.49 (0.90–2.40)	0.11

*HDL* high-density lipoprotein *BMI* body mass index, *BP* blood pressure, *OR* odds ratio, *CI* confidence interval

Model 1, crude model

Model 2 adjusted for age, gender, education level, marital status

Model 3 adjusted for model 2 plus smoking, alcohol consumption, tea consumption, dietary pattern, sleep quality

## Discussion

Depressive symptoms were not related to MetS in the present sample of Chinese community-dwelling male and female older adults. However, we found that depressive symptoms were associated with MetS components including DBP and FPG and diabetes. In general, the study findings were consistent with the results in a recent meta-analysis of observational studies, which demonstrated that the risk of developing diabetes mellitus in depressive individuals was 1.41 times higher than non-depressive ones [23]. Similar associations were found in a longitudinal study with British elders [24]. In addition, a study of Chinese population also demonstrated that FPG concentration was positively associated with the prevalence of depressive symptoms [25]. Shared pathophysiological mechanisms may exist in depressive symptoms and diabetes such as visceral obesity [26], decreased adiponectin level [27] and cortisol dysregulation from the hypothalamic–pituitary–adrenal axis [28]. These pathophysiological mechanisms may be complicated with depressive symptoms and lead to insulin resistance and diabetes.

No associations were found between depressive symptoms and MetS as well as MetS components among women, while significant inverse associations were found among men. Different from our study, Scuteri and colleagues showed that women but not men were more likely to have decreased nocturnal SBP when depression was present [29]. Regardless of gender, the relationship between depression and blood pressure is not confirmative. Two cross-sectional studies showed that depressed elders tended to have lower SBP [30] [31]. A prospective study also found that depression was associated with low BP at 22 years of follow-up [32]. However, this finding was not supported by a 4-year follow-up study [33].

The negative association between SBP and depressive symptoms may be explained by several possible mechanisms. First, individuals with depression are more likely to have cardiovascular disease [34, 35] and may take antihypertensive medications to lower BP levels. However, the present study found that the negative association persisted after adjusting the use of antihypertensive medication. Second, the mechanism is related to a somatic-affective symptom-cluster. General weakness, fatigue and inflammatory process caused by chronic low BP may overlap depressive symptoms [30, 36], or potentially induce depressive symptoms. Third, neuropeptide Y might be a possible marker in the relationship between depression and low BP level [37–39].

The null findings among women may due to gender-related differences in the pathogenesis of depression and hormonal factors. Neural mechanisms associated with pathological distortions while experiencing emotion regulation [40]. In addition, estrogens dropped later and less markedly as human age, women may develop cardiovascular diseases later in life [41, 42]. Further studies need to pay attention to the potential gender-related variations in the relationship between depression and cardiometabolic risk factors.

This study has following limitations needed to be noted. First, the cross-sectional design cannot determine the direction of effects between depression symptoms and MetS. Second, participants self-reported the use of antidepressants medications, which was subject to response bias and recall errors. Even though the diagnostic validity of the PHQ-9 was comparable with a clinician-administered instrument [43], participants may have varied capabilities in completing the questionnaire based on their age, education and cognitive function.

## Conclusions

This study showed that depressive symptoms were negatively associated with elevated SBP in older men but not women. For older Chinese adults, especially men, increasing family attention to depressive symptoms, regular screening and timely diagnosis of depression, as well as monitoring blood glucose and BP routinely are important from a public health perspective. Further studies are warranted to examine the causality and the potential bidirectional relationships between depressive symptoms and MetS in older adults.

## Data Availability

The datasets analyzed in this study are available from the corresponding author (Chen-Wei Pan, pcwonly@gmail.com) upon reasonable request.

## Abbreviations

MetS: Metabolic syndrome
BP: Blood pressure
OR: Odds ratio
CI: Confidence interval
PHQ-9: Nine-item Patient Health Questionnaire
BMI: Body mass index
HDL: High-density lipoprotein
FPG: Fasting plasma glucose
SBP: Systolic blood pressure
DBP: Diastolic blood pressure
